# Validity of Diabetes Self-Reports in the Saku Diabetes Study

**DOI:** 10.2188/jea.JE20120221

**Published:** 2013-07-05

**Authors:** Atsushi Goto, Akemi Morita, Maki Goto, Satoshi Sasaki, Motohiko Miyachi, Naomi Aiba, Masayuki Kato, Yasuo Terauchi, Mitsuhiko Noda, Shaw Watanabe

**Affiliations:** 1 Department of Diabetes Research, Diabetes Research Center, National Center for Global Health and Medicine, Tokyo, Japan; 1 国立国際医療研究センター 糖尿病研究センター 糖尿病研究部; 2 National Institute of Health and Nutrition, Tokyo, Japan; 2 国立健康・栄養研究所; 3 Department of Nutrition, College of Nutrition, Koshien University, Takarazuka, Hyogo, Japan; 3 甲子園大学栄養学部栄養学科; 4 Department of Endocrinology and Metabolism, Yokohama City University Graduate School of Medicine, Yokohama, Japan; 4 横浜市立大学大学院医学研究科 分子内分泌・糖尿病内科; 5 Department of Social and Preventive Epidemiology, School of Public Health, University of Tokyo, Tokyo, Japan; 5 東京大学大学院医学系研究科 公共健康医学専攻社会予防疫学分野; 6 Department of Nutrition and Life Science, Kanagawa Institute of Technology, Atsugi, Kanagawa, Japan; 6 神奈川工科大学 応用バイオ科学部 栄養生命科学科; 7 Japan Foundation for the Promotion of International Medical Research Cooperation, Tokyo, Japan; 7 国際協力医学研究振興財団; 8 Department of Diabetes and Metabolic Medicine, Center Hospital, National Center for Global Health and Medicine, Tokyo, Japan; 8 国立国際医療研究センター病院 糖尿病研究連携部

**Keywords:** diabetes, self-reported diabetes, validation study, undiagnosed diabetes

## Abstract

**Background:**

Diabetes is an important risk factor for cardiovascular disease, certain types of cancer, and death, and self-reports are one of the most convenient methods for ascertaining diabetes status. We evaluated the validity of diabetes self-reports among Japanese who participated in a health checkup.

**Methods:**

Self-reported diabetes was cross-sectionally compared with confirmed diabetes among 2535 participants aged 28 to 85 years in the Saku cohort study. Confirmed diabetes was defined as the presence of at least 1 of the following: fasting plasma glucose (FPG) level of 126 mg/dL or higher, 2-hour post-load glucose (2-hPG) level of 200 mg/dL or higher after a 75-gram oral glucose tolerance test, glycated hemoglobin (HbA1c) level of 6.5% or higher, or treatment with hypoglycemic medication(s).

**Results:**

Of the 251 participants with self-reported diabetes, 121 were taking hypoglycemic medication(s) and an additional 69 were classified as having diabetes. Of the 2284 participants who did not self-report diabetes, 80 were classified as having diabetes. These data yielded a sensitivity of 70.4%, a specificity of 97.3%, a positive predictive value of 75.7%, and a negative predictive value of 96.5%. The frequency of participants with undiagnosed diabetes was 3.0%. Of these, 64.2% had FPG within the normal range and were diagnosed by 2-hPG and/or HbA1c.

**Conclusions:**

Our findings provide additional support for the use of self-reported diabetes as a measure of diabetes in epidemiologic studies performed in similar settings in Japan if biomarker-based diagnosis is difficult.

## Abstract (ja)

**背景：:**

糖尿病は心血管疾患、一部の癌、死亡の重要なリスク因子とされているが、質問票による糖尿病の自己申告は糖尿病を把握する際の簡便な方法の１つである。本研究は、健康診断を受診した日本人を対象に、糖尿病自己申告の妥当性を検討することを目的とした。

**方法：:**

対象は佐久コホート研究の参加者2535名(28－85歳)で、糖尿病自己申告の妥当性を横断的に検討した。空腹時血糖値 ≥ 126 mg/dL、75gブドウ糖負荷後2時間血糖値 ≥ 200 mg/dL、HbA1c ≥ 6.5%、血糖降下薬の使用、のいずれかの基準を満たす者を糖尿病と判定した。

**結果：:**

251名が糖尿病を自己申告していた。そのうち、121名が血糖降下薬を使用しており、69名は血糖降下薬を使用していなかったが、空腹時血糖値、75gブドウ糖負荷後2時間血糖値、HbA1c、のいずれかの基準を満たした。糖尿病を自己申告しなかった2284名のうち、80名が空腹時血糖値、75gブドウ糖負荷後2時間血糖値、HbA1c、のいずれかの基準を満たしたため、糖尿病と判定された。その結果、糖尿病自己申告による感度は70.4%、特異度は97.3%であり、陽性反応的中率は75.7%、陰性反応的中率は96.5%であった。また、未診断糖尿病の割合は3.0%であった。未診断糖尿病の64.2%の対象者は空腹時血糖値が正常域であったが、負荷後2時間血糖値、HbA1c、のいずれか基準を満たしたため糖尿病と判定された。

**結論：:**

日本人の集団を対象とした疫学研究において、血液検査を施行することが困難な場合、自己申告により糖尿病を把握することを支持する結果であった。

## INTRODUCTION

A worldwide epidemic of type 2 diabetes is expected,^1^^–^^3^ and its associated complications may increase mortality, decrease quality of life, and threaten national economies.^4^ There is therefore a considerable need for further investigation of the environmental and genetic determinants of type 2 diabetes. Recent technologic advances have provided opportunities to conduct epidemiologic studies investigating a variety of factors—including genomics, proteomics, and metabolomics—related to diabetes risk and prevalence. Further, diabetes has been recognized as an important risk factor for cardiovascular disease,^5^ certain types of cancer,^6^ and death.^7^ Ascertainment of diabetes is necessary in such epidemiologic studies, and self-reported diabetes is one of the most convenient methods of doing so. Although every effort should be made to obtain biomarker-based confirmation of diabetes, several previous prospective cohort studies have used self-reported diabetes as an outcome when self-administered questionnaires regarding diabetes history were available but laboratory findings were not.^8^^–^^10^ However, few studies have investigated the validity of diabetes self-reports.^8^^,^^9^^,^^11^^,^^12^ The aim of this study was to evaluate the validity of self-reported diabetes among Japanese who participated in a health checkup.

## METHODS

### Study population

This was a cross-sectional analysis of participants in the Saku cohort study, which was launched in 2009 at the Saku General Hospital Human Dock Center in Saku city, Nagano prefecture, Japan. Participants who visited the center for a voluntary health checkup between May 5, 2009 and September 30, 2010 and who agreed to participate were included in the study. From the study population of individuals aged 28 to 85 years at baseline (*n* = 2565), we excluded 30 subjects with missing data. All the remaining 2535 participants had been tested for fasting plasma glucose (FPG) and glycated hemoglobin (HbA1c) levels and were included in the current analysis. Of these participants, 251 responded positively to the question, “Have you been diagnosed as having diabetes mellitus?” (the questionnaire was originally in Japanese). In addition, 121 of these 251 participants reported that they were taking hypoglycemic medication(s). Furthermore, a 75-gram oral glucose tolerance test (75-g OGTT) was performed for 2319 participants (103 of the 251 with self-reported diabetes and 2216 who did not self-report diabetes).

This study was reviewed and approved by the Ethical Committee of the National Institute of Health and Nutrition and by Saku Central Hospital. Participants received a precise explanation of the study and provided their written informed consent.

### Diagnosis of diabetes

According to the recommendation of the Japan Diabetes Society (JDS),^13^ diabetes was confirmed when at least 1 of the following was present: a FPG level of 126 mg/dL or higher, a 2-hour post-load glucose (2-hPG) level of 200 mg/dL or higher after a 75-g OGTT, an HbA1c level of 6.5% or higher, or treatment with hypoglycemic medication(s).

### Laboratory procedures

After an overnight fast, blood samples were collected during the health checkup at the Saku Health Dock Center. Blood samples were collected in tubes containing EDTA and heparin for measurement of FPG and HbA1c levels. HbA1c levels were measured using high-performance liquid chromatography (Tosoh HLC-723 G8; Tosoh Corporation, Tokyo, Japan), with intra- and inter-assay coefficients of variation (CVs) of 0.5% to 1.4% and 0.6% to 1.3%, respectively. The HbA1c values were recorded as JDS values and then converted to National Glycohemoglobin Standardization Program (NGSP) values by using the following conversion formula: HbA1c (NGSP) = 1.02 × HbA1c (JDS) + 0.25%.^14^ Plasma glucose levels were analyzed using an enzymatic method (ECO glucose buffer; A&T Corporation, Kanagawa, Japan), with intra- and inter-assay CVs of 0.3% to 0.5% and 0.6% to 0.8%, respectively.

### Statistical analysis

To examine the validity of self-reported diabetes in this study, we computed sensitivity, specificity, positive predictive value, and negative predictive value. The 95% CIs for the results were determined by the binomial exact method using SAS (version 9.3; SAS institute, Cary, NC, USA).

## RESULTS

The average (SD) age of the 2535 participants (1534 men and 1001 women) was 59.3 (9.6), and average body mass index was 23.1 (3.1). A total of 461 (18.2%) had a family history of diabetes. Table 1 shows the distribution of participants according to laboratory findings and use of hypoglycemic medication. Of the 251 participants with self-reported diabetes, 121 were taking hypoglycemic medication(s), and an additional 69 were classified as having diabetes according to JDS criteria.^13^ Table 2 shows the validity of diabetes self-reports in this study. The positive predictive value was 75.7% (95% CI, 69.9–80.9). Of the 2284 participants who did not self-report diabetes, 80 were classified as having diabetes, yielding a negative predictive value of 96.5% (95% CI, 95.7–97.2). The specificity and sensitivity of self-reported diabetes for identifying diabetes were 97.3% (95% CI, 96.6–97.9) and 70.4% (95% CI, 64.5–75.8), respectively. Stratification according to sex showed that sensitivity was slightly higher among women than among men. Sensitivity and specificity were 69.4% (95% CI, 62.4–75.7) and 97.2% (95% CI, 96.1–98.0), respectively, among men and 73.2% (95% CI, 61.4–83.1) and 97.5% (95% CI, 96.3–98.4), respectively, among women. In addition, after excluding a subgroup of subjects who neither reported hypoglycemic medication use nor underwent the 75-g OGTT, our study population showed similar results: a sensitivity of 71.2% (95% CI, 65.0–77.0) and a specificity of 97.5% (95% CI, 96.7–98.1). When we excluded participants who reported hypoglycemic medication use, sensitivity and specificity were 46.3% (95% CI, 45.4–61.9) and 97.3% (95% CI, 96.6–97.9), respectively, and positive and negative predictive values were 46.9% (95% CI, 44.1–61.9) and 96.5 (95% CI, 95.7–97.2), respectively.

**Table 1.** Distribution of participants according to laboratory findings and hypoglycemic medication use in the Saku diabetes study (*n* = 2535)

**Self-reported diabetes with hypoglycemic medication(s) (*n* = 121)**			
(i) FPG ≥ 126 mg/dL	(ii) 2-hPG ≥ 200 mg/dL	(iii) HbA1c ≥ 6.5%	(iv) Any of (i)–(iii)

63.6%	0.0%	66.9%	81.0%
(77/121)	(0/2)	(81/121)	(98/121)

**Self-reported diabetes without hypoglycemic medications (%) (*n* = 130)**			
(i) FPG ≥ 126 mg/dL	(ii) 2-hPG ≥ 200 mg/dL	(iii) HbA1c ≥ 6.5%	(iv) Any of (i)–(iii)

26.2%	25.7%	30.0%	53.1%
(34/130)	(26/101)	(39/130)	(69/130)

**No self-reported diabetes (*n* = 2284)**			
(i) FPG ≥ 126 mg/dL	(ii) 2-hPG ≥ 200 mg/dL	(iii) HbA1c ≥ 6.5%	(iv) Any of (i)–(iii)

1.6%	2.2%	1.1%	3.5%
(37/2284)	(49/2216)	(24/2284)	(80/2284)

Abbreviations: FPG, fasting plasma glucose; 2-hPG, 2-hour post-load glucose.

**Table 2.** Validity of self-reported diabetes in the Saku diabetes study (*n* = 2535)

	Diabetes^a^	No diabetes	Total (*n*)
Self-reported diabetes (*n*)	190	61	251
No self-reported diabetes (*n*)	80	2204	2284
Total (*n*)	270	2265	2535

**Validity of self-reported diabetes in identifying diabetes**^a^			
	Point estimate	95% CI	

Sensitivity	70.4%	64.5–75.8%	
Specificity	97.3%	96.6–97.9%	
Positive predictive value	75.7%	69.9–80.9%	
Negative predictive value	96.5%	95.7–97.2%	

^a^Diabetes was confirmed when at least 1 of the following was present: a fasting plasma glucose level of 126 mg/dL or higher, a 2-hour post-load glucose level of 200 mg/dL or higher, an HbA1c level of 6.5% or higher, or treatment with hypoglycemic medication(s).

Sensitivity (%) = 100 × (true positives)/(true positives + false negatives).

Specificity (%) = 100 × (true negatives)/(true negatives + false positives).

Positive predictive value (%) = 100 × (true positives)/(true positives + false positives).

Negative predictive value (%) = 100 × (true negatives)/(true negatives + false negatives).

The 95% CIs for the results were determined by the binomial exact method.

In addition, laboratory findings were used to determine the proportion of undiagnosed diabetes patients from among those who did not self-report diabetes and underwent 75-g OGTT (*n* = 2216). On the basis of a combination of FPG, 2-hPG, and HbA1c screenings, 67 participants (3.0%) were classified as having undiagnosed diabetes (Table 3). Screening for HbA1c alone identified 85.1% (*n* = 57/67) of participants with undiagnosed diabetes, screening for FPG alone identified 35.8% (*n* = 24/67), and screening for 2-hPG alone identified 73.1% (*n* = 49/67) (Figure). Screening for the combination of HbA1c and FPG identified 94.0% (*n* = 63/67) of participants with undiagnosed diabetes, but screening for the combination of HbA1c and 2-hPG identified all participants with undiagnosed diabetes (Figure).

**Figure.**
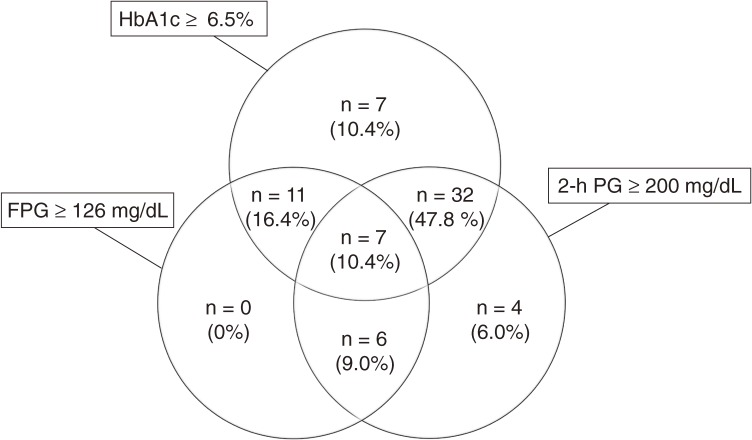
Proportion of participants with undiagnosed diabetes according to fasting plasma glucose, 2-h post-load glucose, and HbA1c (*n* = 67) levels. Abbreviations: FPG, fasting plasma glucose; 2-hPG, 2-hour post-load glucose.

**Table 3.** Distribution of participants with undiagnosed diabetes according to laboratory findings (*n* = 67)

		*n*
**FPG ≥ 126 mg/dL and 2-hPG ≥ 200 mg/dL**		
and HbA1c ≥ 6.5%	10.4%	7
and HbA1c < 6.5%	9.0%	6
**FPG ≥ 126 mg/dL and 2-hPG < 200 mg/dL**		
and HbA1c ≥ 6.5%	16.4%	11
and HbA1c < 6.5%	0%	0
**FPG < 126 mg/dL and 2-hPG ≥ 200 mg/dL**		
and HbA1c ≥ 6.5%	47.8%	32
and HbA1c < 6.5%	6.0%	4
**FPG < 126 mg/dL and 2-hPG < 200 mg/dL**		
and HbA1c ≥ 6.5%	10.4%	7

Total		67

On the basis of a combination of FPG, 2-hPG, and HbA1c screenings, 67 participants were classified as having undiagnosed diabetes.

Diabetes was confirmed when at least 1 of the following was present: a fasting plasma glucose level of 126 mg/dL or higher, a 2-hour post-load glucose level of 200 mg/dL or higher, an HbA1c level of 6.5% or higher, or treatment with hypoglycemic medication(s).

Abbreviations: FPG, fasting plasma glucose; 2-hPG, 2-hour post-load glucose.

## DISCUSSION

In this cross-sectional analysis of Japanese adults who underwent a health checkup, the validity of self-reported diabetes was reasonably high. Although the sensitivity of this method in identifying diabetes was not perfect, possibly owing to undiagnosed diabetes, the sufficiently high specificity and reasonably high sensitivity of self-reported diabetes for identifying diabetes lend support to the use of self-reported diabetes in epidemiologic studies performed in similar settings in Japan.

Our findings are similar to those of previous validation studies on self-reported diabetes in Japan. In the Japan Collaborative Cohort Study for Evaluation of Cancer Risk (JACC Study), self-report data were compared with laboratory findings (fasting serum glucose concentration ≥140 mg/dL or a randomly measured concentration ≥200 mg/dL) and treatment status in a sample of 1230 men and 1837 women. In the JACC study, the sensitivity of self-reported diabetes was 80% for men and 75% for women; specificity was 95% for men and 98% for women.^8^ Also, in the Japan Public Health Centre-based prospective Study (JPHC Study), a medical record review confirmed 94% of self-reported diabetes cases; among the 5927 subjects who did not self-report diabetes, 49 (0.83%) had diabetes as defined by an FPG level of 140 mg/dL or higher, yielding a sensitivity of 82.9% and a specificity of 99.7%.^11^ The sensitivity of self-reported diabetes in our study (70.4%) was lower than that in these previous studies, suggesting that the use of the 75-g OGTT in about 97% of the participants who did not self-report diabetes minimized the possibility of missing diabetes cases. Indeed, the combination of 2-hPG and HbA1c results identified all participants with undiagnosed diabetes in our study.

Several other validation studies of self-reported diabetes have been conducted in Asia, Europe, and the United States.^12^^,^^15^^–^^19^ In the Nurses’ Health Study,^12^ the Health Professional Follow-up Study,^15^ and the Women’s Health Study,^16^ all of which examined the validity of self-reported diabetes among health professionals, medical record reviews confirmed greater than 96% of self-reported diabetes cases. In a validation study conducted among participants in the Women’s Health Initiative (WHI) trials who never self-reported diabetes, there was no medical record or laboratory evidence of diabetes in 94.5% (95% CI, 89.9–97.2) of the participants, and medical record reviews in the WHI confirmed 91.8% (95% CI, 87.0–95.0) of self-reports of diabetes.^17^ Further, in a recent analysis of data from the Atherosclerosis Risk in Communities (ARIC) Study, self-reported diabetes was found to have 84% to 97% specificity and 55% to 80% sensitivity as compared with multiple reference definitions.^19^ The findings of the WHI and ARIC studies support the view that self-reported diabetes is a valid outcome in pragmatic clinical trials and observational studies even among populations of non-health professionals.

Importantly, our findings suggest that the validity of diabetes self-reports might be relatively low among participants who do not use hypoglycemic medications; after excluding participants with hypoglycemic medications, the sensitivity decreased from 70.4% to 46.3% and the positive predictive value decreased from 75.7% to 46.9%. Because the reproducibility of FPG and OGTT results in individuals is limited,^13^ confirmation of diabetes based on a single test—without a medical record review—possibly underestimated the number of patients with diabetes.

Our findings are also consistent with previous validation studies of self-reported hypertension,^20^^,^^21^ hyperlipidemia,^21^ and hyperuricemia.^21^ In the National Integrated Project for Prospective Observation of Non-communicable Diseases and its Trends in the Aged, 1980 (NIPPON DATA80), the sensitivity and specificity of self-reported hypertension for confirmative hypertension were 52% to 65% and 95%, respectively.^20^ The researchers also found that self-reported hypertension was associated with cardiovascular disease mortality.^20^ These findings suggest that self-reports of diseases such as diabetes and hypertension may have relatively low sensitivity and high specificity but can be useful for identifying individuals with diabetes or hypertension if laboratory findings or blood pressure measurements are difficult to obtain.

If laboratory findings cannot be obtained, but self-administered questionnaires regarding diabetes history are available, self-reported diabetes may be used as an outcome. Regarding the impact of bias due to self-reported diabetes on relative risk, with almost perfect specificity, nondifferential misclassification of the outcome has little impact on relative risk measures^22^; however, in cases of differential misclassification the magnitude and direction of bias introduced by outcome misclassification should be carefully evaluated. The specificity of self-reported diabetes in our study was fairly high in all scenarios, which supports the use of self-reported diabetes as a measure of diabetes status.

We found that the frequency of participants with undiagnosed diabetes was 3.0%. The Funagata study, published 20 years ago, documented a relatively high frequency of undiagnosed diabetes (4.9%) in the Funagata area, a rural area in Yamagata Prefecture.^23^ The frequency was lower (3.0%) in our study conducted in Saku city, a rural area in Nagano Prefecture. This suggests that the frequency of undiagnosed diabetes in Japan may be decreasing over time. Our study population was recruited from individuals who participated voluntarily in health checkups and some thus might have been screened for diabetes several times previously. The prevalence of undiagnosed diabetes might be low among such a population. A regional difference in frequency may also explain the difference between studies in the frequency of undiagnosed diabetes. Of note, the frequency of undiagnosed diabetes in our study is comparable to that (2.9%) in the National Health and Nutrition Survey, which was conducted among the general Japanese population.^24^ Also, the frequency of hypoglycemic medication use among the present participants defined as having diabetes (49%) was close to that (46%) in the survey,^24^ which supports the generalizability of our findings. In addition, screening for FPG alone identified only 35.8% of participants with undiagnosed diabetes, but screening for HbA1c alone or 2-hPG alone identified 85.1% and 73.1% of participants, respectively. Further, combined screening of HbA1c and 2-hPG identified 100%. Importantly, earlier epidemiologic studies (eg, the Funagata study) reported that post-challenge hyperglycemia was associated with a higher risk of macrovascular complications.^25^^,^^26^ Taken together, these findings indicate that a diagnosis based on FPG alone might overlook people with diabetes who are at higher risk for macrovascular complications.

The strengths of this study include its large sample size and use of 75-g OGTT and HbA1c levels. Combined use of 75-g OGTT and HbA1c levels minimized the possibility of including undiagnosed diabetes in the true-negative group. Despite its strengths, our study has some limitations. First, because we did not perform a medical record review, we may have underestimated the number of true positives and overestimated the number of true negatives, which might have biased our estimates. Second, we used a single 75-g OGTT to screen participants, which raises the possibility that some participants were misclassified as confirmed diabetes cases due to day-to-day variations in glucose levels or dietary conditions on the test day. When we classified confirmed diabetes cases according to hypoglycemic medication use or HbA1c level only, sensitivity increased (87.0%; 95% CI, 81.2–91.5) but specificity did not change (96.1%; 95% CI, 95.3–96.9). Third, we did not have any information on the frequency of diabetes screening. Finally, it should be noted that when self-reported diabetes is used to identify type 2 diabetes, the age range of the study population must be carefully considered.

In conclusion, in this validation study in a Japanese population, self-reported diabetes was found to be a valid measure of diabetes. Although efforts should be made to obtain laboratory findings to identify participants with diabetes, our findings provide supportive evidence for the use of self-reported diabetes in epidemiologic studies performed under similar settings in Japan when biomarker-based diagnosis is difficult. The findings also suggest that HbA1c and 2-hPG are both important, but that FPG alone may not be useful in identifying undiagnosed diabetes.

## ONLINE ONLY MATERIALS

Abstract in Japanese.
