# Supplementary material for: Validity of Diabetes Self-Reports in the Saku Diabetes Study
Source: J Epidemiol. 2013 Jul 5;23(4):295–300. doi: 10.2188/jea.JE20120221 (PMC3709549; doi:10.2188/jea.JE20120221)
Supplement: Abstract in Japanese. [file je-23-295-s001.pdf]

佐久糖尿病研究における糖尿病自己申告の妥当性

後藤温[1,2,4]、森田明美[2,3]、後藤麻貴[1,2,4]、佐々木敏[5]、宮地元彦[2]、饗場直美[6]、加藤昌之[7]、寺内康夫[4]、野田光彦[1,8]、渡邊昌[2]、佐久コホート研究グループ
[1]国立国際医療研究センター　糖尿病研究センター　糖尿病研究部
[2]国立健康・栄養研究所
[3]甲子園大学栄養学部栄養学科
[4]横浜市立大学大学院医学研究科　分子内分泌・糖尿病内科
[5]東京大学大学院医学系研究科　公共健康医学専攻社会予防疫学分野
[6]神奈川工科大学　応用バイオ科学部　栄養生命科学科
[7]国際協力医学研究振興財団
[8]国立国際医療研究センター病院　糖尿病研究連携部

背景：糖尿病は心血管疾患、一部の癌、死亡の重要なリスク因子とされているが、質問票による糖尿病の自己申告は糖尿病を把握する際の簡便な方法の 1 つである。本研究は、健康診断を受診した日本人を対象に、糖尿病自己申告の妥当性を検討することを目的とした。
方法：対象は佐久コホート研究の参加者 2535 名(28－85 歳)で、糖尿病自己申告の妥当性を横断的に検討した。空腹時血糖値 ≥126 mg/dL、75g ブドウ糖負荷後 2 時間血糖値 ≥200 mg/dL、HbA1c ≥ 6.5%、血糖降下薬の使用、のいずれかの基準を満たす者を糖尿病と判定した。
結果：251 名が糖尿病を自己申告していた。そのうち、121 名が血糖降下薬を使用しており、69 名は血糖降下薬を使用していなかったが、空腹時血糖値、75g ブドウ糖負荷後 2 時間血糖値、HbA1c、のいずれかの基準を満たした。糖尿病を自己申告しなかった 2284 名のうち、80 名が空腹時血糖値、75g ブドウ糖負荷後 2 時間血糖値、HbA1c、のいずれかの基準を満たしたため、糖尿病と判定された。その結果、糖尿病自己申告による感度は 70.4%、特異度は 97.3%であり、陽性反応的中率は 75.7%、陰性反応的中率は 96.5%であった。また、未診断糖尿病の割合は 3.0%であった。未診断糖尿病の 64.2%の対象者は空腹時血糖値が正常域であったが、負荷後 2 時間血糖値、HbA1c、のいずれか基準を満たしたため糖尿病と判定された。
結論：日本人の集団を対象とした疫学研究において、血液検査を施行することが困難な場合、自己申告により糖尿病を把握することを支持する結果であった。

キーワード：
糖尿病、糖尿病自己申告、妥当性研究、未診断糖尿病
